# *N*′-(4-Hydroxy­benzyl­idene)aceto­hydrazide monohydrate

**DOI:** 10.1107/S160053680902892X

**Published:** 2009-07-29

**Authors:** Lu-Ping Lv, Tie-Ming Yu, Wen-Bo Yu, Wei-Wei Li, Xian-Chao Hu

**Affiliations:** a Department of Chemical Engineering, Hangzhou Vocational and Technical College, Hangzhou 310018, People’s Republic of China; b Research Center of Analysis and Measurement, Zhejiang University of Technology, Hangzhou 310014, People’s Republic of China

## Abstract

In the title compound, C_9_H_10_N_2_O_2_·H_2_O, the mol­ecular skeleton of the acetohydrazide mol­ecule is nearly planar [within 0.014 (1) Å]. The mol­ecule adopts a *trans* configuration with respect to the C=N bond, while the side chain is slightly twisted away from the attached ring, forming a dihedral angle of 9.975 (8)°. The crystal packing exhibits a three-dimensional network composed from alternating acetohydrazide mol­ecules and uncoordinated water mol­ecules, which inter­act *via* N—H⋯O, O—H⋯O and O—H⋯N hydrogen bonds. A C—H⋯π inter­action is also present.

## Related literature

For general background to the analytical applications of Schiff bases, see: Ciemerman *et al.* (1997 ▶). For their mild bacteriostatic activity and potential use as oral iron-chelating drugs for the treatment of genetic disorders such as thalassemia, see: Offe *et al.* (1952 ▶); Richardson *et al.* (1988 ▶). For a related structure, see: Li & Jian (2008 ▶); Tamboura *et al.* (2009 ▶).
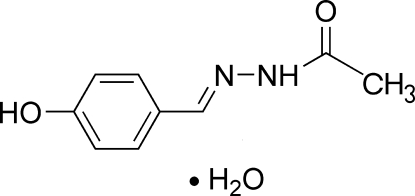


## Experimental

### 

#### Crystal data

C_9_H_10_N_2_O_2_·H_2_O
*M* *_r_* = 196.21
Monoclinic, 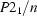

*a* = 8.352 (2) Å
*b* = 10.146 (3) Å
*c* = 12.328 (3) Å
β = 105.353 (3)°
*V* = 1007.3 (5) Å^3^
*Z* = 4
Mo *K*α radiation
μ = 0.10 mm^−1^
*T* = 223 K
0.23 × 0.21 × 0.20 mm

#### Data collection

Bruker SMART CCD area-detector diffractometer
Absorption correction: multi-scan (*SADABS*; Bruker, 2002 ▶) *T*_min_ = 0.969, *T*_max_ = 0.976
4820 measured reflections
1764 independent reflections
1569 reflections with *I* > 2σ(*I*)
*R*_int_ = 0.015

#### Refinement

*R*[*F* ^2^ > 2σ(*F* ^2^)] = 0.037
*wR*(*F* ^2^) = 0.100
*S* = 1.06
1764 reflections
147 parameters
H atoms treated by a mixture of independent and constrained refinement
Δρ_max_ = 0.18 e Å^−3^
Δρ_min_ = −0.22 e Å^−3^

### 

Data collection: *SMART* (Bruker, 2002 ▶); cell refinement: *SAINT* (Bruker, 2002 ▶); data reduction: *SAINT*; program(s) used to solve structure: *SHELXS97* (Sheldrick, 2008 ▶); program(s) used to refine structure: *SHELXL97* (Sheldrick, 2008 ▶); molecular graphics: *SHELXTL* (Sheldrick, 2008 ▶); software used to prepare material for publication: *SHELXTL*.

## Supplementary Material

Crystal structure: contains datablocks I, global. DOI: 10.1107/S160053680902892X/bg2278sup1.cif

Structure factors: contains datablocks I. DOI: 10.1107/S160053680902892X/bg2278Isup2.hkl

Additional supplementary materials:  crystallographic information; 3D view; checkCIF report

## Figures and Tables

**Table 1:** Hydrogen-bond geometry (Å, °)

*D*—H⋯*A*	*D*—H	H⋯*A*	*D*⋯*A*	*D*—H⋯*A*
O1—H1⋯O2^i^	0.82	2.00	2.7477 (15)	152
N2—H2*A*⋯O1*W*^ii^	0.86	1.96	2.8060 (17)	166
O1*W*—H1*F*⋯O2	0.88 (2)	1.92 (2)	2.7600 (17)	159 (2)
O1*W*—H1*E*⋯O1^iii^	0.85 (2)	2.01 (2)	2.8241 (17)	161 (2)
O1—H1⋯N1^i^	0.82	2.54	3.1864 (16)	137
C9—H9*B*⋯*Cg*1^iv^	0.96	2.74	3.519 (2)	138

Symmetry codes: (i) 
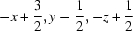
; (ii) 
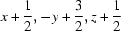
; (iii) 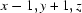; (iv) 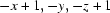. *Cg*1 is the centroid of the C1–C6 ring.

**Fig. 1.**
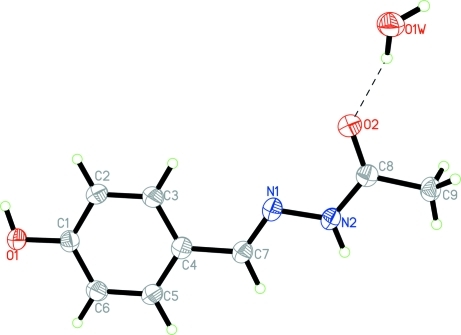
The asymmetric unit of the title compound. Displacement ellipsoids are drawn at the 40% probability level. Dashed lines indicate hydrogen bonds.

**Fig. 2.**
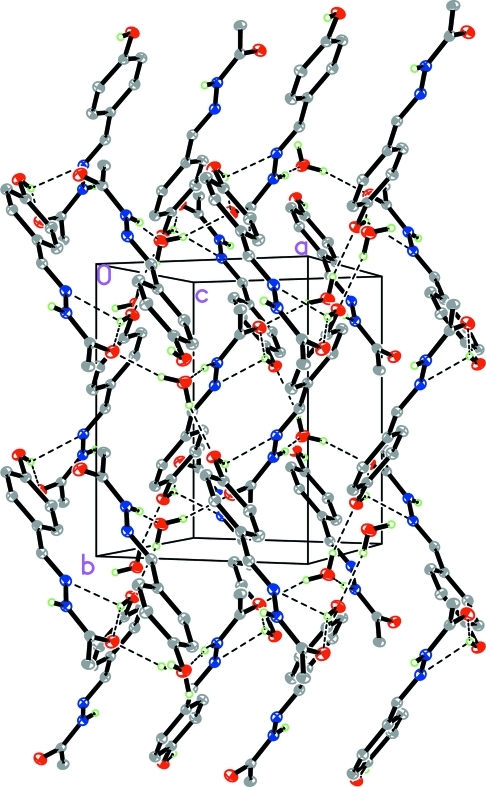
Crystal packing of the title compound. Hydrogen bonds are shown as dashed lines.

## supplementary crystallographic information

### Comment

Schiff bases have attracted much attention due to their possibility of analytical application (Ciemerman *et al.*, 1997). They are also important ligands, which have been reported to have mild bacteriostatic activity and potential oral iron-chelating drugs for genetic disorders such as thalassemia (Offe *et al.*, 1952, Richardson *et al.*, 1988). Metal complexes based on Schiff bases have received considerable attention because they can be utilized as model compounds of active centres in various complexes (Tamboura *et al.*, 2009). We report here the crystal structure of the title compound (Fig. 1).

In the title compound,C_9_H_10_N_2_O_2_ .H_2_O, (I) the molecular skeleton  is nearly planar. The molecule  adopts a trans configuration with respect to the C═N bond, while the side chain is slightly twisted away from the attached ring. The dihedral angle between these two essentially planar units is 9.975 (8)°. Bond lengths and angles are comparable to those observed for *N*'-[1-(4-methoxyphenyl)ethylidene]acetohydrazide (Li *et al.*, 2008).

The crystal packing exhibits a three-dimensional network composed from alternating molecules of (I) and crystalline water, which interact via N-H···O, O-H···O and O-H···N hydrogen bonds. In addition, a intermolecular C—H···π interactions is observed (Table 1 and Fig 2).

### Experimental

4-Hydroxybenzaldehyde (1.22 g, 0.01 mol) and acetohydrazide (0.74 g, 0.01 mol) were dissolved in stirred methanol (20 ml) and left for 2.5 h at room temperature. The resulting solid was filtered off and recrystallized from ethanol to give the title compound in 95% yield. Single crystals suitable for X-ray analysis were obtained by slow evaporation of an ethanol solution at room temperature (m.p. 435–437 K).

### Refinement

H atoms of the water molecule were located in a difference map and were refined with O-H distances restrained to 0.84 (2) Å and 0.88 (2) Å, Other H atoms were positioned geometrically (N-H = 0.86 Å , O-H=0.82Å and C-H = 0.93 or 0.96Å) and refined using a riding model, with *U*_iso_(H) =1.2*U*_eq_(C,N) and 1.5*U*_eq_(C_methyl_). In the absence of significant anomalous scattering effects, Friedel pairs were averaged.

### Crystal data

**Table:**

C_9_H_10_N_2_O_2_·H_2_O	*F*(000) = 416
*M**_r_* = 196.21	*D*_x_ = 1.294 Mg m^−^^3^
Monoclinic, *P*2_1_/*n*	Mo *K*α radiation, λ = 0.71073 Å
Hall symbol: -P 2yn	Cell parameters from 1764 reflections
*a* = 8.352 (2) Å	θ = 2.6–25.0°
*b* = 10.146 (3) Å	µ = 0.10 mm^−^^1^
*c* = 12.328 (3) Å	*T* = 223 K
β = 105.353 (3)°	Block, colourless
*V* = 1007.3 (5) Å^3^	0.23 × 0.21 × 0.20 mm
*Z* = 4	

### Data collection

**Table:**

Bruker SMART CCD area-detector diffractometer	1764 independent reflections
Radiation source: fine-focus sealed tube	1569 reflections with *I* > 2σ(*I*)
graphite	*R*_int_ = 0.015
φ and ω scans	θ_max_ = 25.0°, θ_min_ = 2.6°
Absorption correction: multi-scan (*SADABS*; Bruker, 2002)	*h* = −9→9
*T*_min_ = 0.969, *T*_max_ = 0.976	*k* = −10→12
4820 measured reflections	*l* = −14→14

### Refinement

**Table:**

Refinement on *F*^2^	Primary atom site location: structure-invariant direct methods
Least-squares matrix: full	Secondary atom site location: difference Fourier map
*R*[*F*^2^ > 2σ(*F*^2^)] = 0.037	Hydrogen site location: inferred from neighbouring sites
*wR*(*F*^2^) = 0.100	H atoms treated by a mixture of independent and constrained refinement
*S* = 1.06	*w* = 1/[σ^2^(*F*_o_^2^) + (0.0527*P*)^2^ + 0.2585*P*] where *P* = (*F*_o_^2^ + 2*F*_c_^2^)/3
1764 reflections	(Δ/σ)_max_ < 0.001
147 parameters	Δρ_max_ = 0.18 e Å^−^^3^
0 restraints	Δρ_min_ = −0.22 e Å^−^^3^

### Special details

**Table:**

Geometry. All esds (except the esd in the dihedral angle between two l.s. planes) are estimated using the full covariance matrix. The cell esds are taken into account individually in the estimation of esds in distances, angles and torsion angles; correlations between esds in cell parameters are only used when they are defined by crystal symmetry. An approximate (isotropic) treatment of cell esds is used for estimating esds involving l.s. planes.
Refinement. Refinement of F^2^ against ALL reflections. The weighted R-factor wR and goodness of fit S are based on F^2^, conventional R-factors R are based on F, with F set to zero for negative F^2^. The threshold expression of F^2^ > 2sigma(F^2^) is used only for calculating R-factors(gt) etc. and is not relevant to the choice of reflections for refinement. R-factors based on F^2^ are statistically about twice as large as those based on F, and R- factors based on ALL data will be even larger.

### Fractional atomic coordinates and isotropic or equivalent isotropic displacement parameters (Å^2^)

**Table:**

	*x*	*y*	*z*	*U*_iso_*/*U*_eq_	Occ. (<1)
C1	0.96500 (16)	0.24160 (13)	0.32475 (11)	0.0320 (3)	
C2	0.86072 (18)	0.34460 (14)	0.27501 (11)	0.0376 (3)	
H2	0.8381	0.3587	0.1979	0.046 (4)*	
C3	0.79116 (17)	0.42545 (14)	0.33983 (12)	0.0370 (3)	
H3	0.7221	0.4941	0.3060	0.046 (4)*	
C4	0.82307 (16)	0.40561 (13)	0.45606 (11)	0.0319 (3)	
C5	0.92557 (17)	0.30058 (14)	0.50398 (11)	0.0348 (5)	0.998 (6)
H5	0.9473	0.2853	0.5809	0.040 (4)*	
C6	0.99522 (17)	0.21901 (13)	0.43931 (11)	0.0352 (3)	
H6	1.0623	0.1490	0.4725	0.041 (4)*	
C7	0.75676 (16)	0.49358 (13)	0.52741 (11)	0.0337 (3)	
H7	0.7852	0.4793	0.6047	0.041 (4)*	
C8	0.51412 (16)	0.77123 (13)	0.52939 (11)	0.0321 (3)	
C9	0.47333 (19)	0.85289 (14)	0.61982 (12)	0.0408 (4)	
H9A	0.5276	0.8165	0.6921	0.094 (7)*	
H9B	0.3554	0.8528	0.6100	0.104 (8)*	
H9C	0.5109	0.9416	0.6153	0.086 (7)*	
N1	0.66075 (13)	0.58964 (11)	0.48641 (9)	0.0333 (3)	
N2	0.61346 (14)	0.66803 (11)	0.56438 (9)	0.0329 (3)	
H2A	0.6479	0.6504	0.6350	0.045 (4)*	
O1	1.04051 (13)	0.16176 (10)	0.26415 (8)	0.0421 (3)	
H1	1.0135	0.1844	0.1979	0.080 (7)*	
O2	0.45976 (13)	0.79883 (10)	0.42813 (8)	0.0436 (3)	
O1W	0.17157 (17)	0.90361 (12)	0.29134 (10)	0.0527 (3)	
H1E	0.146 (3)	0.984 (2)	0.2989 (18)	0.075 (7)*	
H1F	0.260 (3)	0.886 (2)	0.3465 (19)	0.078 (7)*	

### Atomic displacement parameters (Å^2^)

**Table:**

	*U*^11^	*U*^22^	*U*^33^	*U*^12^	*U*^13^	*U*^23^
C1	0.0344 (7)	0.0292 (7)	0.0343 (7)	−0.0020 (5)	0.0123 (6)	−0.0026 (5)
C2	0.0447 (8)	0.0401 (8)	0.0294 (7)	0.0043 (6)	0.0122 (6)	0.0036 (6)
C3	0.0396 (7)	0.0355 (7)	0.0367 (7)	0.0061 (6)	0.0117 (6)	0.0040 (6)
C4	0.0313 (7)	0.0317 (7)	0.0338 (7)	−0.0048 (5)	0.0106 (5)	−0.0023 (5)
C5	0.0381 (8)	0.0378 (9)	0.0289 (7)	−0.0024 (6)	0.0096 (6)	0.0011 (6)
C6	0.0372 (7)	0.0315 (7)	0.0366 (7)	0.0026 (6)	0.0091 (6)	0.0035 (6)
C7	0.0343 (7)	0.0361 (7)	0.0314 (7)	−0.0043 (6)	0.0101 (5)	−0.0026 (5)
C8	0.0319 (7)	0.0313 (7)	0.0350 (7)	−0.0061 (5)	0.0121 (6)	−0.0004 (5)
C9	0.0474 (9)	0.0345 (8)	0.0435 (8)	−0.0027 (6)	0.0173 (7)	−0.0066 (6)
N1	0.0350 (6)	0.0347 (6)	0.0324 (6)	−0.0024 (5)	0.0129 (5)	−0.0048 (5)
N2	0.0363 (6)	0.0352 (6)	0.0281 (6)	−0.0004 (5)	0.0103 (5)	−0.0035 (4)
O1	0.0545 (7)	0.0391 (6)	0.0353 (6)	0.0119 (5)	0.0164 (5)	−0.0007 (4)
O2	0.0534 (6)	0.0448 (6)	0.0349 (5)	0.0090 (5)	0.0155 (5)	0.0055 (4)
O1W	0.0697 (8)	0.0430 (7)	0.0384 (6)	0.0116 (6)	0.0019 (6)	−0.0018 (5)

### Geometric parameters (Å, °)

**Table:**

C1—O1	1.3644 (16)	C7—H7	0.9300
C1—C6	1.3864 (19)	C8—O2	1.2421 (17)
C1—C2	1.3941 (19)	C8—O2	1.2421 (17)
C2—C3	1.377 (2)	C8—N2	1.3346 (18)
C2—H2	0.9300	C8—C9	1.4988 (19)
C3—C4	1.4009 (19)	C9—H9A	0.9600
C3—H3	0.9300	C9—H9B	0.9600
C4—C5	1.3962 (19)	C9—H9C	0.9600
C4—C7	1.4611 (19)	N1—N2	1.3832 (16)
C5—C6	1.380 (2)	N2—H2A	0.8600
C5—H5	0.9300	O1—H1	0.8200
C6—H6	0.9300	O1W—H1E	0.85 (2)
C7—N1	1.2782 (18)	O1W—H1F	0.88 (2)
			
O1—C1—C6	118.28 (12)	N1—C7—H7	119.1
O1—C1—C2	121.98 (12)	C4—C7—H7	119.1
C6—C1—C2	119.74 (12)	O2—C8—N2	122.15 (12)
C3—C2—C1	120.12 (12)	O2—C8—N2	122.15 (12)
C3—C2—H2	119.9	O2—C8—C9	121.90 (13)
C1—C2—H2	119.9	O2—C8—C9	121.90 (13)
C2—C3—C4	120.85 (13)	N2—C8—C9	115.95 (12)
C2—C3—H3	119.6	C8—C9—H9A	109.5
C4—C3—H3	119.6	C8—C9—H9B	109.5
C5—C4—C3	118.15 (12)	H9A—C9—H9B	109.5
C5—C4—C7	119.94 (12)	C8—C9—H9C	109.5
C3—C4—C7	121.88 (12)	H9A—C9—H9C	109.5
C6—C5—C4	121.22 (12)	H9B—C9—H9C	109.5
C6—C5—H5	119.4	C7—N1—N2	115.36 (11)
C4—C5—H5	119.4	C8—N2—N1	119.60 (11)
C5—C6—C1	119.90 (13)	C8—N2—H2A	120.2
C5—C6—H6	120.1	N1—N2—H2A	120.2
C1—C6—H6	120.1	C1—O1—H1	109.5
N1—C7—C4	121.72 (12)	H1E—O1W—H1F	107 (2)
			
O1—C1—C2—C3	−177.94 (13)	C2—C1—C6—C5	−1.7 (2)
C6—C1—C2—C3	1.5 (2)	C5—C4—C7—N1	−179.03 (12)
C1—C2—C3—C4	−0.2 (2)	C3—C4—C7—N1	3.0 (2)
C2—C3—C4—C5	−0.8 (2)	C4—C7—N1—N2	−177.28 (11)
C2—C3—C4—C7	177.21 (13)	O2—C8—N2—N1	1.12 (19)
C3—C4—C5—C6	0.6 (2)	O2—C8—N2—N1	1.12 (19)
C7—C4—C5—C6	−177.44 (12)	C9—C8—N2—N1	−178.27 (11)
C4—C5—C6—C1	0.6 (2)	C7—N1—N2—C8	179.57 (12)
O1—C1—C6—C5	177.77 (12)		

### Hydrogen-bond geometry (Å, °)

**Table:**

*D*—H···*A*	*D*—H	H···*A*	*D*···*A*	*D*—H···*A*
O1—H1···O2^i^	0.82	2.00	2.7477 (15)	152
N2—H2A···O1W^ii^	0.86	1.96	2.8060 (17)	166
O1W—H1F···O2	0.88 (2)	1.92 (2)	2.7600 (17)	159 (2)
O1W—H1E···O1^iii^	0.85 (2)	2.01 (2)	2.8241 (17)	161 (2)
O1—H1···N1^i^	0.82	2.54	3.1864 (16)	137
C9—H9B···Cg1^iv^	0.96	2.74	3.519 (2)	138

Symmetry codes: (i) −*x*+3/2, *y*−1/2, −*z*+1/2; (ii) *x*+1/2, −*y*+3/2, *z*+1/2; (iii) *x*−1, *y*+1, *z*; (iv) −*x*+1, −*y*, −*z*+1.
